# Circulating Tumor DNA as a Prognostic Biomarker in Localized Non-small Cell Lung Cancer

**DOI:** 10.3389/fonc.2020.561598

**Published:** 2020-09-15

**Authors:** Muyun Peng, Qi Huang, Wei Yin, Sichuang Tan, Chen Chen, Wenliang Liu, Jingqun Tang, Xiang Wang, Bingyu Zhang, Min Zou, Jina Li, Wenhui Su, Lientu Wang, Lihan Chin, Fenglei Yu

**Affiliations:** ^1^Department of Thoracic Surgery, The Second Xiangya Hospital of Central South University, Changsha, China; ^2^Hunan Key Laboratory of Early Diagnosis and Precise Treatment of Lung Cancer, The Second Xiangya Hospital of Central South University, Changsha, China; ^3^Department of Biomedical Sciences, Chang Gung Molecular Medicine Research Center, Graduate Institute of Biomedical Sciences, College of Medicine, Chang Gung University, Taoyuan City, Taiwan; ^4^Department of Otolaryngology, Chang Gung Memorial Hospital, Linkou, Taiwan; ^5^Berry Oncology Co., Ltd., Beijing, China

**Keywords:** non-small cell lung cancer, circulating tumor DNA, circulating single molecule amplification and re-sequencing technology, prognostic biomarker, minimal residual disease

## Abstract

**Background:**

Routine clinical surveillance involves serial radiographic imaging following radical surgery in localized non-small cell lung cancer (NSCLC). However, such surveillance can detect only macroscopic disease recurrence and is frequently inconclusive. We investigated if detection of ctDNA before and after resection of NSCLC identifies the patients with risk of relapse, and furthermore, informs about response to management.

**Methods:**

We recruited a total of 77 NSCLC patients. A high-throughput 127 target-gene capture technology and a high-sensitivity circulating single-molecule amplification and resequencing technology (cSMART) assay were used to detect the somatic mutations in the tumor tissues as well as the plasma of NSCLC patients before and after surgery to monitor for minimal residual disease (MRD). Kaplan-Meier and Cox regression analysis were performed to evaluate the relapse-free survival (RFS) and overall survival (OS) of patients with predictor variables.

**Results:**

Patients with a higher stage (III/IV) and preoperative ctDNA-positive status demonstrated a significant 2.8-3.4-fold risk and 3.8-4.0-fold risk for recurrence and death, respectively. Preoperative ctDNA-positive patients associated with a lower RFS (HR = 3.812, *p* = 0.0005) and OS (HR = 5.004, *p* = 0.0009). Postoperative ctDNA-positive patients also associated with a lower RFS (HR = 3.076, *p* = 0.0015) and OS (HR = 3.195, *p* = 0.0053). Disease recurrence occurred among 63.3% (19/30) of postoperative ctDNA-positive patients. Most of these patients 89.5% (17/19) had detectable ctDNA within 2 weeks after surgery and was identified in advance of radiographic findings by a median of 12.6 months.

**Conclusion:**

Advanced stage and preoperative ctDNA-positive are strong predictors of RFS and OS in localized NSCLC patients undergoing complete resection. Postoperative detection of ctDNA increases chance to detect early relapse, thus can fulfill an important role in stratifying patients for immediate further treatment with adjuvant and neoadjuvant therapy.

## Introduction

Lung cancer is a leading cause of cancer-related mortality, with an estimated nearly 2.1 million new cancer cases diagnosed leading to an estimated 1.8 million deaths worldwide in 2018 (1). In the United States, it is estimated that approximately 230,000 new cases of lung cancer will be diagnosed, and about 140,000 people will die from the disease in 2019 (2). For early-stage non-small cell lung cancer (NSCLC) patients, the best chance for cure is a complete (R0) resection by anatomic lung resection with mediastinal lymph node dissection. The 5-year overall survival (OS) of this approach is 61.5% and 5-year recurrence-free survival (RFS) is 59.0% (3). Following a radical surgery, serial radiographic imaging is routine. Because such surveillance detects only macroscopic recurrence, it is frequently inconclusive due to postoperative normal tissue changes. Given the population health burden of lung cancer, there is an imperative to develop a sensitive and specific biomarker that can detect the molecular residual disease (MRD) before macroscopic recurrence.

Plasma circulating tumor DNA (ctDNA) are DNA fragments in the blood that contain tumor-specific somatic alterations. It can be collected at minimal discomfort to the patient. The detection of ctDNA is a promising strategy for the prognosis and surveillance of solid tumors (4–6). It can predict the recurrence of non-metastatic breast, colon and pancreas cancers (7–9). Likewise, it can be detected in the postoperative blood sample in 94% of lung patients experiencing recurrence, and the results precede radiographic findings by a median of 5.2 months in 72% of patients (10). Being able to identify microscopic remnants of tumor cells and metastases can drastically change treatment algorithms, especially for localized lung cancer. In this study, we set out to determine whether preoperative or postoperative ctDNA positive status can predict survival outcomes in patients with localized lung cancer.

Furthermore, the detection of a positive EGFR mutation in the plasma of NSCLC patient during third cycle of treatment by erlotinib or chemotherapy has been associated with a reduced progression-free survival (PFS) and OS (11). We also addressed the hypothesis that post-surgical ctDNA detection could guide personalized interventions of adjuvant and neoadjuvant therapy.

## Materials and Methods

### Study Cohort

A total of 81 patients diagnosed with solitary lung nodules intended for surgery at the Thoracic Surgery Department of The Second Xiangya Hospital were collected into our study between February 2014 and December 2015. All patients gave their written informed consent for specimen collection, provision of clinical information, and biomarker analysis before they participated in the study. The study was conducted in accordance with the Declaration of Helsinki, and the protocol was approved by the Ethics Committee of the Second Xiangya Hospital, Central South University, Changsha (Project identification code: 2014S006).

### Study Inclusion Criteria

Eligible patients were age >18 years old with solitary lung nodules who agreed to the curative-intent treatment in this study. Preoperatively, the patients received chest computer tomography (CT) scans. Their blood samples were collected 1–7 days before surgery. Postoperatively, the patients received surveillance CT scans or positron emission tomography (PET)-CT scans at a series of scheduled time-points (2 weeks, 3, 6, 12, 18, and 24 months). Blood samples were also collected from patients during these follow-up visits. Patients with stage II or higher, if their physical condition permits, were further treated by adjuvant chemotherapy or target therapy (with or without radiotherapy) 1 month after surgery. If a recurrence or metastasis was suspected, a biopsy was performed to confirm the diagnosis. If a biopsy was not possible, then surgery, chemotherapy, radiotherapy or targeted therapy would be administered according to the specific situation.

### Pathological Analysis of Tumor Specimens

Tumor specimens were collected by surgery. Macro-dissection was performed to enrich the tumor tissue percentage to around 80% before DNA extraction. Histologic evaluation of stained FFPE tumor sections was used to confirm the diagnosis of NSCLC. For the clinical staging of disease, the criteria from the TNM staging system of the International Association for the Study of Lung Cancer (version 8) was used.

### DNA Isolation From FFPE and Plasma Samples

Genomic DNA and total RNA Isolation from FFPE specimen blocks or scrapings from cytological slides were performed using the AmoyDx FFPE DNA and RNA Kits with nucleic acid purification spin columns (Amoy Diagnostics, Xiamen, China). By spectroscopy analysis, all purified DNA and RNA samples were verified to be of high quality for mutation analysis. Matching 10-mL blood samples, collected in Streck tubes (Streck, La Vista, NE, United States), was taken by venipuncture within 48 h of tumor specimen collection. DNA for the cSMART assay was prepared from 2 mL of purified plasma using a commercially available kit (QIAamp DNA Blood MiniKit, Qiagen, Hilden, Germany). The concentration of the purified DNA was measured by the Qubit^®^ dsDNA HS Assay Kit (Life Technologies, Grand Island, NY, United States).

### cSMART Plasma Assay

A novel cSMART assay (12, 13) was used for the detection and quantitation of hotspot oncogenic ctDNA mutations targeting 127 recurrent mutations in lung cancer (Supplementary Table S1). In brief, 50 ng of FFPE DNA was fragmented in NEB Next dsDNA fragmentase buffer (New England Biolabs, MA, United States) to an average size of 300 bp. DNA libraries were generated as previously described (14) except that a degenerate 4 bp barcode sequence was incorporated into the sequencing adaptor for uniquely identifying and counting single allelic molecules. Single DNA molecules were circularized and targeted with back-to-back primers located within 20 to 48 bp from the mutation loci to ensure maximum sensitivity and specificity for mutation detection. The ctDNA status was classified as detectable (ctDNA-positive) or undetectable (ctDNA-negative) according to mutation ratio. A mutation ratio equal to 0 is defined as ctDNA-negative and greater than 0 is defined as ctDNA-positive.

### Statistical Analyses

Data were summarized using descriptive statistics. The ctDNA variables in the different groups were compared using the Kruskal–Wallis non-parametric test. Fisher’s exact test was used to test for the association between ctDNA detection and histological subtypes. The primary outcome measure was RFS as evaluated by standard RECIST criteria. RFS was defined as the length of time after surgery that the patient survives without any signs or symptoms of lung cancer. OS was defined as the length of time from the date of diagnosis until death. Curves for RFS and OS were constructed using the Kaplan–Meier method and compared using the log-rank test. Multivariable Cox proportional hazards regression analyses for RFS and OS were performed using the Wald test to assess the predictive ability of preoperative and postoperative ctDNA. Analyses were performed using statistical software Graphpad Prism (version 7.0) and SPSS (version 22). Power calculation for Cox proportional hazards regression was estimated using the R package “powerSurvEpi^1^.” We used the powerEpi function that takes into account the correlation between two covariates, which we considered to be stage and preoperative ctDNA status. For RFS and OS, taking a minimum postulated hazard ratio (HR) of 2.7 and 3.7, power estimates were 69 and 77%, respectively, at a type I error rate of 0.05.

## Results

### Patient Characteristics and Tissue Capture

**FIGURE 1:**
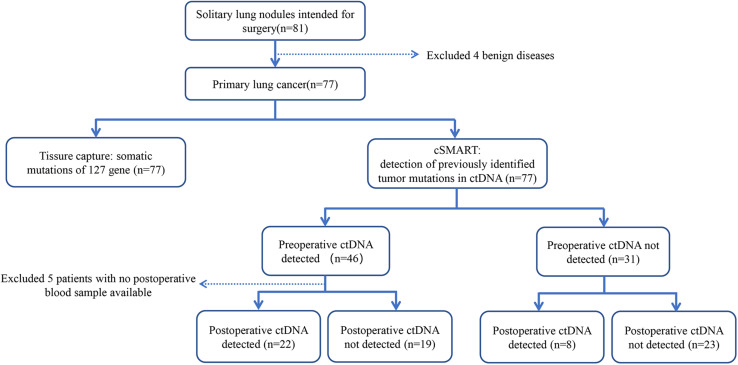
Consort diagram of patient enrollment, specimen collection and clinical management.

After accounting for missing data, 77 patients underwent surgery for localized lung cancer were included in the final analysis (Figure 1). We collected a total of 77 tissue samples. Accompanying this was the collection of 77 preoperative blood samples that belonged to 77 patients and 199 postoperative blood samples drawn at different times from 71 patients (Supplementary Table S2).

Table 1 and Supplementary Table S3 summarizes the clinical characteristics of patients. Their median age was 60.3 years old, have a male to female ratio of 2.7 and most were never smokers (49, 63.6%). Those diagnosed with adenocarcinoma, squamous carcinoma, and others, the numbers were 40 (51.9%), 30 (39.0%), and 7 (9.1%) patients, respectively. Those with tumors located in the right-upper, right-middle, right-lower, left-upper, and left -lower lobes, the numbers were 26 (33.8%), 2 (2.6%), 18 (23.3%), 20 (26.0%), and 11 (14.3%) patients, respectively. Those diagnosed with disease stages I, II, III, and IV, the numbers were 41 (53.2%), 18 (23.4%), 16 (20.8%), and 2 (2.6%) patients, respectively. The two cases of stage IV patients were accidentally found to have pleural implant metastasis during operation.

**TABLE 1:** The clinical characteristics of patients.

Clinical parameters	Patients (*N* = 77)
Age (years)	60.3 (40∼78)
**Gender**	
Male	56 (72.7%)
Female	21 (27.3%)
BMI (kg/m^2^)	23.4
**Smoking history**	
Never smoker	49 (63.6%)
Ever smoker	28 (36.4%)
**Tumor position**	
Central	23 (29.9%)
Peripheral	54 (70.1%)
**Pathology**	
Lung adenocarcinoma	40 (51.9%)
Lung squamous carcinoma	30 (39.0%)
Others	7 (9.1%)
**Tumor stage**	
Stage I	40 (51.9%)
Stage II	18 (23.4%)
Stage III	17 (22.1%)
Stage IV	2 (2.6%)
**Lobe**	
Right-upper	26 (33.8%)
Right-middle	2 (2.6%)
Right-lower	18 (23.3%)
Left-upper	20 (26.0%)
Left-lower	11 (14.3%)

*Data are given as *N* (percentage) unless otherwise indicated. *N*, number; BMI, body mass index.*

All the 77 patients harbored at least one mutation in their tumor tissue, with an average of 1.2 gene mutations per patient (Supplementary Table S4). The most frequent mutations observed in tissues located in the *TP53* (60%), *EGFR* (21%), *KEAP1* (9%), *NAV3* (8%), *CDKN2A* (8%), and *PIK3CA* (8%) genes (Supplementary Figure S1).

### Preoperative and Postoperative ctDNA Status

**FIGURE 2:**
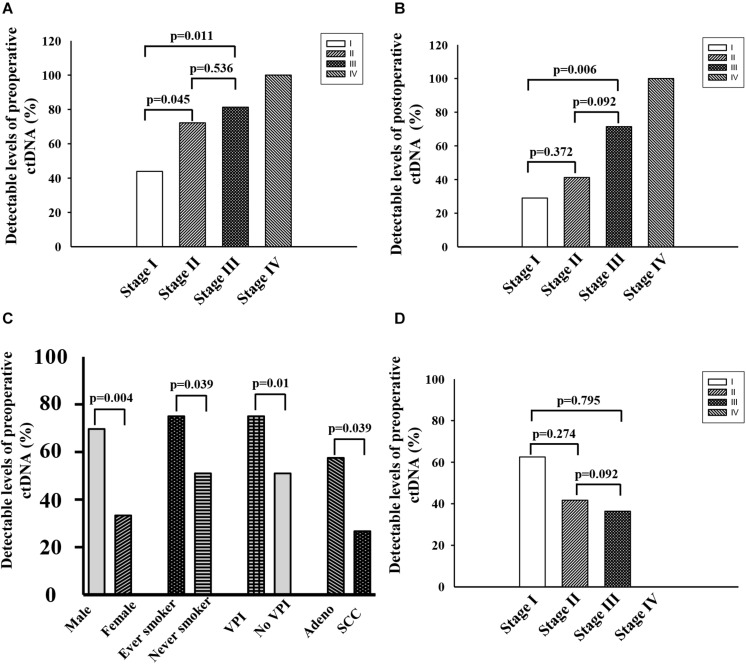
**(A)** Fraction of patients with detectable preoperative ctDNA in NSCLC at cancer stage I∼IV. **(B)** Fraction of patients with detectable postoperative ctDNA in NSCLC at cancer stage I∼IV. **(C)** Characteristics associated with positive preoperative ctDNA. **(D)** Negative conversion ratio of preoperative ctDNA at cancer stage I∼III.

Preoperative ctDNA-positive status was detected in 46 of 77 patients (59.7%). They constituted 43.9% (18/41), 72.2% (13/18), 81.3% (13/16), and 100% (2/2) of patients with stage I, II, III, and IV, respectively (Figure 2A). They further associated with males, never smokers, lung squamous carcinoma and visceral pleural invasion (*p* < 0.01) (Figure 2C). There was no association between preoperative ctDNA-positive status and age or BMI. Among 46 patients with positive preoperative ctDNA, the average number of gene mutation is 1.46, with one gene mutation detected in 29 cases, 2 in 13 cases, and 3 in 4 cases (Supplementary Table S2).

Postoperative ctDNA-positive status was detected in 30 of 71 patients (42.25%). They formed 29.0% (11/38), 41.2% (7/17), 71.4% (10/14), and 100.0% (2/2) of patients with stage I, II, III, and IV, respectively (Figure 2B). Among the 41 patients with detectable preoperative ctDNA, 22 of them continued to have detectable postoperative ctDNA. The negative conversion ratios were 62.50% (10/16), 41.67% (5/12), 36.36% (4/11), and 0.0% (0/2) of patients with stage I, II, III, and IV, respectively (Figure 2D).

### Preoperative and Postoperative ctDNA-Positive Statuses Associated With Lower Recurrence-Free Survival (RFS) and Overall Survival (OS)

**FIGURE 3:**
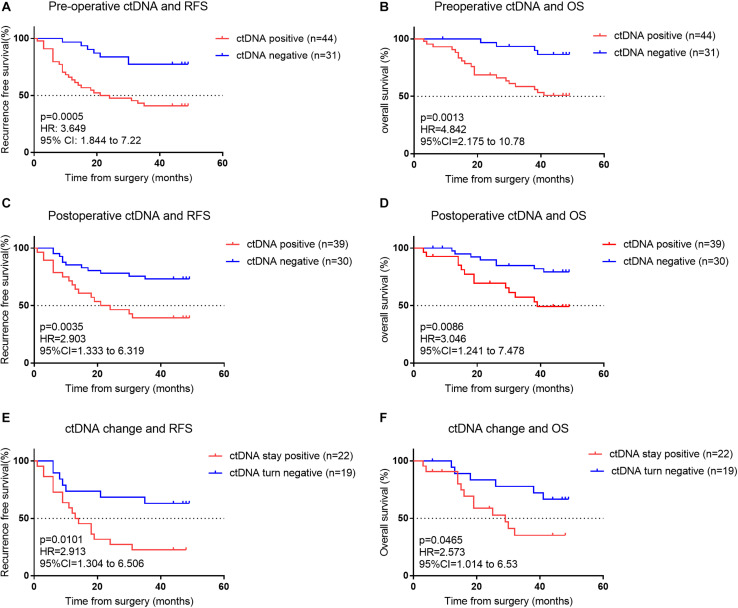
**(A)** Kaplan–Meier estimates of recurrence-free survival (RFS) for all assessable patients undergoing curative intent surgery for early NSCLC cancer, stratified by pre-operative ctDNA status: detectable (positive) versus undetectable (negative). **(B)** Kaplan–Meier estimate for overall survival (OS) for matched patients, stratified by pre-operative ctDNA status. **(C)** Kaplan–Meier estimates for RFS, stratified by postoperative ctDNA status. **(D)** Kaplan–Meier estimates for OS, stratified by post-operative ctDNA status. **(E,F)** Kaplan–Meier estimates for RFS **(E)** and OS **(F)** for patients’ whose ctDNA status changes from detectable (positive) pre-operatively to undetectable (negative) post-operatively compared with patients whose ctDNA status remains undetectable (negative) both pre- and post-operatively.

During the median follow-up period of 46 months in this study, 35 (45%) patients had a cancer recurrence, and 25 (32.47%) patients died from cancer recurrence/metastasis. In stage I–III patients, those with preoperative ctDNA-positive status had a lower RFS and OS than those who did not (*P* < 0.001; Figures 3A,B and Supplementary Tables S2, S5). During the follow-up, patients with postoperative ctDNA-positive status were detected in at least a one-time point for 28 (39%) patients. Among these, 17 of 28 (61%) patients experienced a recurrence, and 13 of them eventually died due to recurrence/metastasis. In stage I–III patients, those with postoperative ctDNA-negative status had a higher RFS and OS than ctDNA-positive patients (*P* < 0.05; Figures 3C,D and Supplementary Tables S2, S5).

We investigated patients whose ctDNA turned negative after surgery, indicating their lesion was completely removed. Among the 41 patients who were preoperative ctDNA-positive, 22 continued to have detectable postoperative ctDNA. Patients with undetectable postoperative ctDNA (ctDNA turned negative) had a significantly better RFS and OS than detectable postoperative ctDNA (ctDNA stayed positive) (*p* < 0.05; Figures 3E,F and Supplementary Tables S2, S5). Postoperative ctDNA preceded the radiographic findings or clinical symptoms by a median of 12.6 months in 19 (63.3%) of patients. In these 19 patients, 17 were postoperative ctDNA-positive within 2 weeks after surgery, implying ctDNA was not fully cleared after surgery and they likely had an MRD. We also found that among the 31 patients who were preoperative ctDNA-negative, 8 have newly detectable postoperative ctDNA.

### Cox Regression Analysis for RFS and OS in Patients With NSCLC

Independent univariate analysis showed that stage, preoperative and postoperative ctDNA statuses were significant clinicopathological factors for RFS and OS (Table 2). After multivariate analysis, only stage and preoperative ctDNA status remained significant for RFS and OS (*p* < 0.05). The results indicated that having a higher stage (III/IV) compared to a lower stage (I/II) conferred a 2.8- or 3.8-fold risk of recurrence/metastasis or death, respectively. Also, having a preoperative ctDNA-positive status, compared to ctDNA-negative status, conferred a 3.4- or 4.0-fold risk of recurrence/metastasis or death, respectively.

**TABLE 2:** Recurrence-free survival (RFS) and overall survival (OS) analysis by clinicopathologic variables and pre- and post-operative ctDNA status.

		Univariate analysis		Multivariate analysis	
Variable and RFS/OS	Num	HR (95% CI)	*p*	HR (95% CI)	*p*
**Variable and RFS**					
Gender, male vs. female	56 vs. 21	0.534 (0.233–1.225)	0.139		
Age, <=60 vs. >60	38 vs. 39	1.126 (0.579–2.190)	0.728		
Smoker, ever vs. never	28 vs. 49	0.329 (0.363–1.405)	0.714		
BMI, <25 vs. >=25	55 vs. 22	0.532 (0.232–1.219)	0.136		
Sidedness of primary tumor, left vs. Right	31 vs. 46	1.362 (0.678–2.738)	0.386		
Position, peripheral vs. central	54 vs. 23	2.014 (1.022–3.972)	0.043		0.173
Pathology, adenocarcinoma vs. SCC	40 vs. 30	0.674 (0.313–1.450)	0.313		
Visceral invasion, present vs. absent	28 vs. 49	0.542 (0.279–1.053)	0.071		0.816
Stage, I/II vs. III/IV	59 vs. 18	3.491 (1.764–6.912)	0.000	2.759 (1.301–5.851)	0.008
Preoperative ctDNA status, negative vs. positive	42 vs. 35	3.858 (1.681–8.855)	0.001	3.401 (1.360–8.507)	0.009
Postoperative ctDNA status, negative vs. positive	30 vs. 41	3.108 (1.474–6.553)	0.003		0.078
**Variable and OS**					
Gender, male vs. female	56 vs. 21	0.693 (0.277–1.738)	0.435		
Age, <=60 vs. >60	38 vs. 39	1.162 (0.527–2.562)	0.709		
Smoker, ever vs. never	28 vs. 49	0.865 (0.382–1.959)	0.728		
BMI, <25 vs. >=25	55 vs. 22	0.524 (0.197–1.397)	0.196		
Sidedness of primary tumor, left vs. Right	31 vs. 46	1.433 (0.618–3.322)	0.401		
Position, peripheral vs. central	54 vs. 23	1.947 (0.873–4.339)	0.103		
Pathology, adenocarcinoma vs. SCC	40 vs. 30	0.671 (0.271–1.665)	0.390		
Visceral invasion, present vs. absent	28 vs. 49	0.525 (0.239–1.151)	0.108		
Stage, I/II vs. III/IV	59 vs. 18	4.578 (2.068–10.138)	0.000	3.784 (1.540–9.300)	0.004
Preoperative ctDNA status, negative vs. positive	42 vs. 35	5.055 (1.731–14.756)	0.003	4.035 (1.346–12.102)	0.013
Postoperative ctDNA status, negative vs. positive	30 vs. 41	3.223 (1.348–7.707)	0.009		0.144

*SCC, squamous cell carcinoma.*

## Discussion

Our findings indicated that among other factors, having a lower disease stage and a preoperative ctDNA-negative status were two strong predictors of a better outcome in RFS and OS. The Kaplan-Meier for RFS and OS stratified by ctDNA detection status also showed that before and after surgery, the ctDNA-negative NSCLC patients had superior RFS and OS compared with ctDNA-positive patients.

We found the ctDNA detection rate dropped from 59.7 to 42.25% after the curative resection of the primary tumor, with most reductions in stage I and II patients. A study of 41 patients tracking ctDNA mutation frequency of 6 tumor driver genes (*EGFR*, *KRAS*, *TP53*, *BRAF*, *PIK3CA*, and *ERBB2*) within 10 days before and after surgical resection, also reported a ctDNA mutation frequency decreased from a median of 8.88 to 0.28%, with most reductions seen in patients with stage I disease (15). In another study of 76 NSCLC patients who underwent curative-intent surgery, the ctDNA mutation frequency decreased from 7.94% ± 4.78% before surgery to 0.28% ± 0.32% after surgery (*p* < 0.001) (16). These results imply that earlier stage lung cancer patients are less likely to have residual disease after resection.

Early-detection strategies have the potential to reduce cancer morbidity and mortality (17). Our previous study have shown that ctDNA can be used in the early diagnosis of lung cancer (18). In this cohort, even for stage I patients, 48.8% of patients have detectable levels of ctDNA in their plasma. In stage III disease, more than four-fifths of patients have detectable ctDNA. Another study has shown ctDNA is detectable in 47% of patients with stage I cancers of any type, and 55, 69, and 82% of patients with stage II, III, and IV cancers, respectively (19).

At present, most solid tumors are treatable by surgery, and even when occult metastasis has occurred, adjuvant therapy or additional surgery can contribute to cure in certain patients (20). Studies are demonstrating the necessity and efficacy of adjuvant and neoadjuvant therapy in early-stage NSCLC patients (21, 22). However, adjuvant chemotherapy in early-stage NSCLC provide an absolute survival benefit of only 4–5% compared to observation or best supportive care (21). Because the current staging model does not accurately identify the molecular residual disease (MRD) or micro-metastases, it could misguide patient selection. Validation of predictive biomarkers is urgently required to facilitate patient selection and risk stratification. Postoperative ctDNA monitoring can help with the identification of patients who could receive the most benefit from neoadjuvant and adjuvant therapy.

Postoperative ctDNA detection of patients correlates with better monitoring of relapse (9). Abbosh et al., report that ctDNA-positive status associated with the relapse of disease after intent-to-cure surgery in NSCLC patients. In that study, 13 of 14 patients experienced relapse had measurable ctDNA before demonstrating clinically evident disease and detection of ctDNA preceded the radiographic diagnosis by a median interval of 70 days. In another study that assessed the MRD in patients with lung cancer using CAPP-Seq, ctDNA was detected after curative-intent therapies among 20 of 37 patients, all of whom had disease recurrence and ctDNA associated with disease relapse earlier than CT imaging by a median lead time of 5.2 months in 72% of lung cancer patients (10). On the contrary, a very few, 1 out of 10 patients, exhibited persistent or recurrent ctDNA levels who did not relapse during a follow-up period of median 775 days (23). The time interval between the postoperative increase in ctDNA levels and the clinical diagnosis of cancer recurrence opens up a window of opportunity for intervention.

Our study focused on the effect of preoperative and postoperative ctDNA status on survival in resectable lung cancer. This study has a relatively long follow-up time, and the conclusion is persuasive. Nevertheless, this article has certain disadvantages. In our cohort, 25 patients received adjuvant therapy, of whom 21 received adjuvant chemotherapy. Because the designed time point of blood collection is not completely coincident with adjuvant chemotherapy, 8 patients lack the comparison before and after chemotherapy, and 7 patients have negative ctDNA before and after adjuvant treatment. Therefore, our data cannot evaluate the impact of adjuvant chemotherapy on ctDNA. However, previous studies have confirmed the possible role of circulating DNA in judging the efficacy of chemotherapy (24, 25). A well-designed clinical trial is needed to verify the role of ctDNA in evaluating the efficacy of neoadjuvant therapy for lung adenocarcinoma.

In summary, when used in conjunction with AJCC staging, ctDNA provides a relatively precise risk stratification. The serial monitoring of ctDNA in a larger cohort of patients receiving neoadjuvant or adjuvant treatment in the future would provide a more robust indication regarding patient selection.

## Data Availability Statement

The raw data supporting the conclusions of this article will be made available by the authors, without undue reservation.

## Ethics Statement

The studies involving human participants were reviewed and approved by the Ethics Committee of the Second Xiangya Hospital, Central South University, Changsha. The patients/participants provided their written informed consent to participate in this study.

## Author Contributions

MP, FY, and LW contributed to the conception and design of the study. MP, QH, WY, ST, CC, WL, JT, XW, BZ, MZ, JL, and FY organized the database. MP, LC, and WS performed the statistical analysis. MP and LC wrote the first draft of the manuscript. All authors contributed to manuscript revision, read, and approved the submitted version.

## Conflict of Interest

LC and LW were employed by the company Berry Oncology Co., Ltd. The remaining authors declare that the research was conducted in the absence of any commercial or financial relationships that could be construed as a potential conflict of interest.

## Footnotes

1 https://cran.r-project.org/web/packages/powerSurvEpi/index.html

## Supplementary Material

FIGURE S1 The most frequently detected mutations in tumor tissue and ctDNA.

TABLE S1 127 gene panel.

TABLE S2 Plasma ctDNA detection indices at each time point for all patients.

TABLE S3 Characteristics of all patients.

TABLE S4 Tissue capture results of all 77 patients.

TABLE S5 Recurrence free survival and overall survival for all patients.
