# Supplementary material for: Circulating Tumor DNA as a Prognostic Biomarker in Localized Non-small Cell Lung Cancer
Source: Front Oncol. 2020 Sep 15;10:561598. doi: 10.3389/fonc.2020.561598 (PMC7523087; doi:10.3389/fonc.2020.561598)
Supplement: FIGURE S1 — The most frequently detected mutations in tumor tissue and ctDNA. [file Presentation_1.PPTX]

## Slide 1
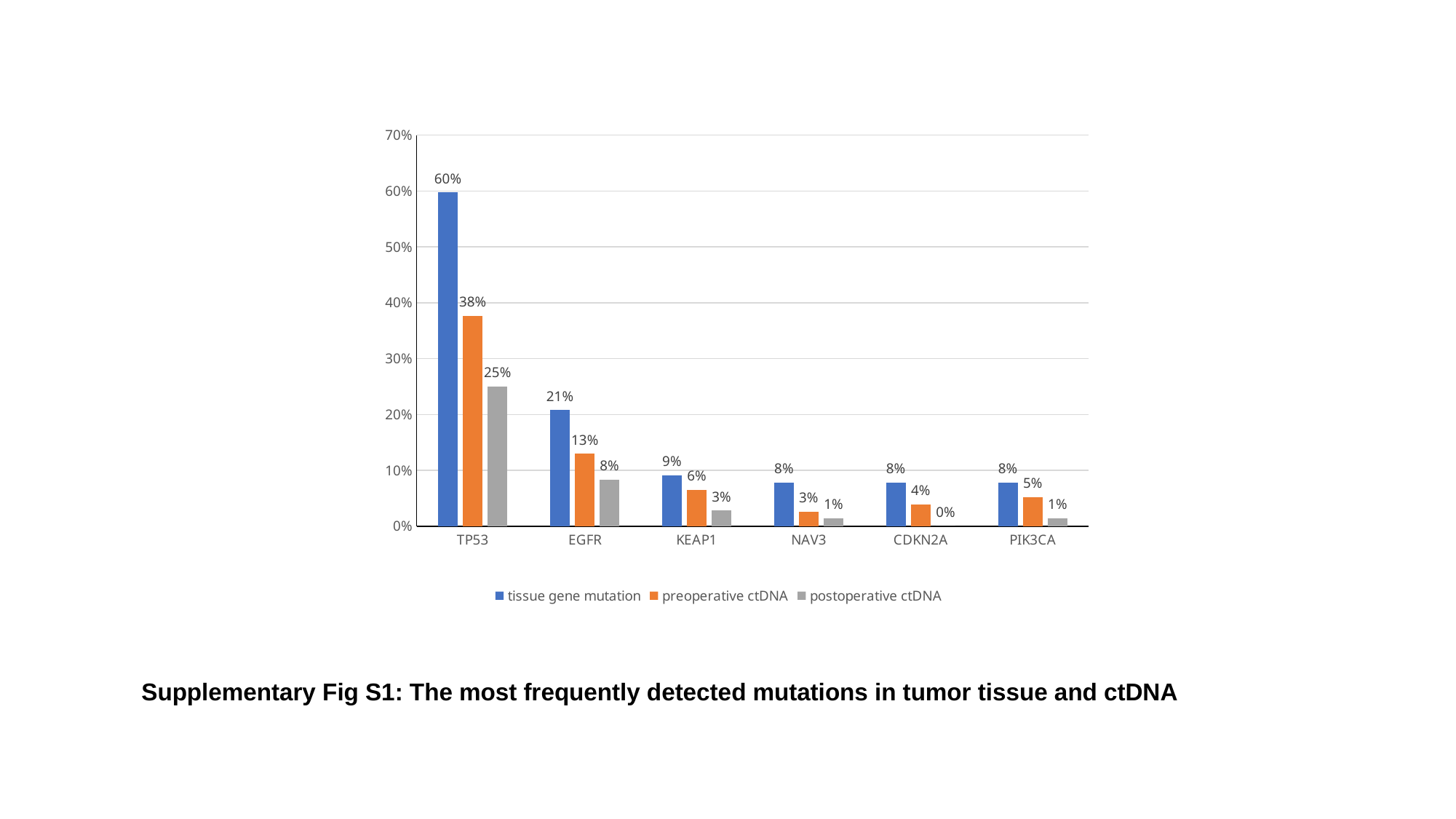

### Chart
| Category | tissue gene mutation | preoperative ctDNA | postoperative ctDNA |
|---|---|---|---|
| TP53 | 0.5974025974025974 | 0.3766233766233767 | 0.25 |
| EGFR | 0.20779220779220786 | 0.12987012987012986 | 0.08333333333333334 |
| KEAP1 | 0.09090909090909094 | 0.06493506493506493 | 0.02777777777777779 |
| NAV3 | 0.07792207792207793 | 0.025974025974025983 | 0.013888888888888892 |
| CDKN2A | 0.07792207792207793 | 0.03896103896103896 | 0.0 |
| PIK3CA | 0.07792207792207793 | 0.05194805194805195 | 0.013888888888888892 |Supplementary Fig S1: The most frequently detected mutations in tumor tissue and ctDNA
